# Supplementary material for: Implementation of an automated scheduling tool improves schedule quality and resident satisfaction
Source: PLoS One. 2020 Aug 11;15(8):e0236952. doi: 10.1371/journal.pone.0236952 (PMC7418963; doi:10.1371/journal.pone.0236952)
Supplement: S1 Fig — (PPTX) [file pone.0236952.s002.pptx]

## Slide 1
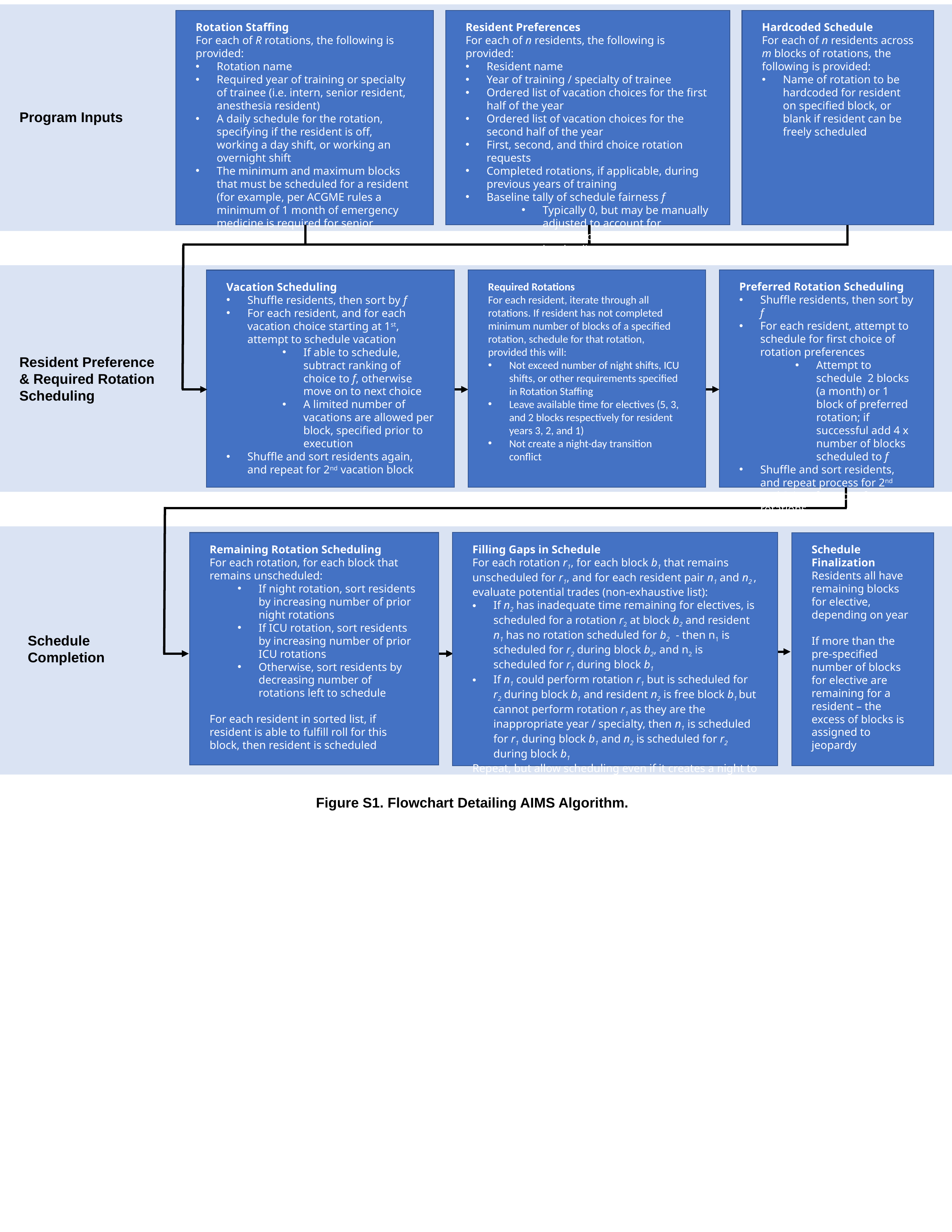

Rotation Staffing
For each of R rotations, the following is provided:
Rotation name
Required year of training or specialty of trainee (i.e. intern, senior resident, anesthesia resident)
A daily schedule for the rotation, specifying if the resident is off, working a day shift, or working an overnight shift
The minimum and maximum blocks that must be scheduled for a resident (for example, per ACGME rules a minimum of 1 month of emergency medicine is required for senior internal medicine residents)
Resident Preferences
For each of n residents, the following is provided:
Resident name
Year of training / specialty of trainee
Ordered list of vacation choices for the first half of the year
Ordered list of vacation choices for the second half of the year
First, second, and third choice rotation requests
Completed rotations, if applicable, during previous years of training
Baseline tally of schedule fairness f
Typically 0, but may be manually adjusted to account for preferences fulfilled by hardcoding
Hardcoded Schedule
For each of n residents across m blocks of rotations, the following is provided:
Name of rotation to be hardcoded for resident on specified block, or blank if resident can be freely scheduled
Program Inputs
Required Rotations
For each resident, iterate through all rotations. If resident has not completed minimum number of blocks of a specified rotation, schedule for that rotation, provided this will:
Not exceed number of night shifts, ICU shifts, or other requirements specified in Rotation Staffing
Leave available time for electives (5, 3, and 2 blocks respectively for resident years 3, 2, and 1)
Not create a night-day transition conflict
Preferred Rotation Scheduling
Shuffle residents, then sort by f
For each resident, attempt to schedule for first choice of rotation preferences
Attempt to schedule 2 blocks (a month) or 1 block of preferred rotation; if successful add 4 x number of blocks scheduled to f
Shuffle and sort residents, and repeat process for 2nd and 3rd preference of rotations
Vacation Scheduling
Shuffle residents, then sort by f
For each resident, and for each vacation choice starting at 1st, attempt to schedule vacation
If able to schedule, subtract ranking of choice to f, otherwise move on to next choice
A limited number of vacations are allowed per block, specified prior to execution
Shuffle and sort residents again, and repeat for 2nd vacation block
Resident Preference & Required Rotation Scheduling
Remaining Rotation Scheduling
For each rotation, for each block that remains unscheduled:
If night rotation, sort residents by increasing number of prior night rotations
If ICU rotation, sort residents by increasing number of prior ICU rotations
Otherwise, sort residents by decreasing number of rotations left to schedule
For each resident in sorted list, if resident is able to fulfill roll for this block, then resident is scheduled
Filling Gaps in Schedule
For each rotation r1, for each block b1 that remains unscheduled for r1, and for each resident pair n1 and n2 , evaluate potential trades (non-exhaustive list):
If n2 has inadequate time remaining for electives, is scheduled for a rotation r2 at block b2 and resident n1 has no rotation scheduled for b2 - then n1 is scheduled for r2 during block b2, and n2 is scheduled for r1 during block b1
If n1 could perform rotation r1 but is scheduled for r2 during block b1 and resident n2 is free block b1 but cannot perform rotation r1 as they are the inappropriate year / specialty, then n1 is scheduled for r1 during block b1 and n2 is scheduled for r2 during block b1
Repeat, but allow scheduling even if it creates a night to day transition conflict, or if number of rotations of a specific type exceeds the cap
Schedule Finalization
Residents all have remaining blocks for elective, depending on year
If more than the pre-specified number of blocks for elective are remaining for a resident – the excess of blocks is assigned to jeopardy
Schedule Completion
Figure S1. Flowchart Detailing AIMS Algorithm.
