# Supplementary material for: Implementation of an automated scheduling tool improves schedule quality and resident satisfaction
Source: PLoS One. 2020 Aug 11;15(8):e0236952. doi: 10.1371/journal.pone.0236952 (PMC7418963; doi:10.1371/journal.pone.0236952)
Supplement: S1 Text — (PDF) [file pone.0236952.s003.pdf]

Scheduling Satisfaction Survey

All questions were answered on a 5 point Likert scale, with 1 indicating least satisfied / least fair, and 5 indicating most satisfied / most fair.

1) How satisfied were you with your schedule *last* year, 2017-2018?

2) How satisfied were you with your schedule *this* year, 2018-2019?

3) How fair was your schedule *last* year, 2017-2018?

4) How fair was your schedule *this* year, 2018-2019?
