# Supplementary material for: Implementation of an automated scheduling tool improves schedule quality and resident satisfaction
Source: PLoS One. 2020 Aug 11;15(8):e0236952. doi: 10.1371/journal.pone.0236952 (PMC7418963; doi:10.1371/journal.pone.0236952)
Supplement: S2 Text — (DOCX) [file pone.0236952.s004.docx]

**Table A. Required Night and ICU Rotations per 2 Week Block by Schedule Year.**

| Mean (SD) | 2017 – 2018 | 2018 - 2019 |
| --- | --- | --- |
| Night Rotations | 14.1 (1.8) | 16.0 (2.6) |
| ICU Rotations | 20.5 (1.9) | 20.6 (2.7) |

**Table B. Intern schedule quality metrics, for manually generated and AIMS generated schedules for the 2017 – 2018 academic year. (*values listed as number per resident per year, SD unless otherwise specified)**

| Mean (SD) | Manual Schedule (n = 72)* | AIMS  (n = 72)* | t-test p-value | F-test p-value |
| --- | --- | --- | --- | --- |
| Night Rotations | 3.7 (0.53) | 3.7 (0.63) | 1.00 | 0.16 |
| ICU Rotations | 3.8 (0.64) | 3.8 (0.59) | 1.00 | 0.53 |
| Jeopardy Rotations | 0.6 (0.57) | 0.6 (0.63) | 0.78 | 0.37 |
| Shift Conflicts | 0.7 (0.90) | 0.0 (0.16) | **< 0.001** |  |
| Average Preference for Assigned Vacations (#, SD) | 1.8 (1.7) | 1.3 (0.50) | **< 0.01** |  |
| Assigned First Choice Rotation (%, SD) | 69.4 (46) | 90.3 (30) | **<0.01** |  |

**Table C. Resident schedule quality metrics, for manually generated and AIMS generated schedules for the 2017 – 2018 academic year. (*values listed as number per resident per year, SD unless otherwise specified)**

| Mean (SD) | Manual Schedule (n = 82)* | AIMS  (n = 82)* | t-test p-value | F-test p-value |
| --- | --- | --- | --- | --- |
| Night Rotations | 1.9 (0.84) | 1.9 (0.87) | 1.00 | 0.76 |
| ICU Rotations | 3.2 (0.53) | 3.2 (0.60) | 1.00 | 0.30 |
| Jeopardy Rotations | 0.7 (0.65) | 0.6 (0.60) | 0.80 | 0.44 |
| Shift Conflicts | 0.1 (35) | 0.0 (20) | **0.01** |  |
| Average Preference for Assigned Vacations (#, SD) | 1.3 (1.3) | 1.4 (0.69) | 0.56 |  |
| Assigned First Choice Rotation (%, SD) | 30.5 (46) | 96.3 (20) | **<0.001** |  |
| Assigned Second Choice Rotation (%, SD) | 46.3 (50) | 76.8 (43) | **<0.001** |  |
