# Supplementary material for: Implementation of an automated scheduling tool improves schedule quality and resident satisfaction
Source: PLoS One. 2020 Aug 11;15(8):e0236952. doi: 10.1371/journal.pone.0236952 (PMC7418963; doi:10.1371/journal.pone.0236952)
Supplement: Attachment — Submitted filename: PONE-D-20-05580-report-20200411.pdf [file pone.0236952.s005.pdf]

Manuscript Number: PONE-D-20-05580

Referee Report on the paper

"Implementation of an Automated Scheduling Tool Improves Schedule Quality and Resident Satisfaction"

Submitted to

PLOS One

Summary and recommendation

This referee report discusses an article about the quality of automating rotation schedules for residents. The authors developed a rotation scheduling tool that was implemented for the Yale New Haven Hospital Internal Medicine Residency Program. In their study, they compare the quality of schedules before and after implementation of the new tool based on objective as well as subjective perception.

Even though the methodical contribution is minor (and not necessary for the journal submitted), the implementation and analysis in a working environment are quite interesting. In general, the paper is very well structured and well written. However, the paper misses a careful literature review. Additionally, the description of the tool/algorithm is only superficial. Based on the following comments, we recommend a major revision.

Major comments

Introduction: The authors jump directly into the topic. Even if you are somewhat familiar with the subject, essential terms should be introduced for the reader (e.g. rotation, jeopardy rotation etc.). A clear problem description might help as well. Please note that residency programs are quite different in various countries. The literature review from line 57-69 is rather short and misses some essential papers in resident scheduling literature (at least from a methodological point of view). We would recommend the literature review "State of the art in physician scheduling" by Erhard et al. (2018) as a starting point for the review. Forward and backward search might be very helpful finding relevant papers.

Method: The description of the scheduling tool is lagging. The process is not entirely transparent at first glance. For instance, you could add a pseudocode to the appendix or use a flow chart for the process. We have a couple of open questions that show the ambiguity or incompleteness of the text.

- What is the time horizon of your schedule?
- Are the problems for residents and interns independent, i.e. you solve both independently? If not, then some explanation in the text is needed.
- What do you mean by scheduling lottery? How often is this lottery performed within the time horizon?
- What kind of preferences do you look at, i.e., only rotation preferences or overnight duties?
- Can this lottery be manipulated, i.e., when I know that I got my first preference in the last lottery, can I change my preferences so that a lower priority might be my true first one? Or are you running the lottery for each 2-week horizon using the data collected for the whole year? It is somewhat unclear in the text.

- What is the “jeopardy” pool? Please explain and add a description (see comment our comments to the introduction).
- A visualization of the algorithm might be helpful.
- What is the objective function of your algorithm, or do you just construct a feasible solution? We assume the latter but it is not clear in the text.
- What are your objective metrics based on, i.e., expert knowledge or literature? We assume the former but again it is not clear in the text. A motivation based on literature might be valuable as well.
- On page 4 you are fixing some input values. What is the effect of relaxing this assumption? We guess you achieve better schedules? But understand that e.g. external rotations are fixed. However, are you planning rotations that are external rotations for other units? Then some coordination between units is necessary. You should motivate the assumption.
- On your tally, what happens if you do not grant any request? We mean: If nothing is fulfilled, then a resident/intern has 0 and is never chosen? How do you initialize the heuristic?
- What happens in case you have less residents/interns available than needed?
- Are you limiting the number of requests of any kind (e.g. preferences, vacations) for each resident/intern?

<u>Results:</u>
We like the comparison between the two years which can be seen as a contribution for itself. However, it might be biased. Just as an idea, you might regenerate schedules for 2017-2018 retrospective and compare the results with the realized ones. Also, it was unclear whether you base your evaluations on the planned or realized (with re-planning) schedules. We think and hope you use planned schedules. If so, the subjective assessment might be biased by re-planning as well. Please make it clear and comment on it in the text. In future, we recommend detailing the subjective questionnaire in alignment with the quality metrics. Can you say something about the savings for the chief resident? E.g. is (s)he faster on top of the quality gains? Also, will the procedure be used next year as well. If so, why? If not so, why? A short outlook might be useful for other practitioners. Just a note from methodological point of view, are you correcting multiple hypotheses testing, or are you just performing single tests in your analysis? For the latter, some of your results might not be significant anymore. You might use a step-wise procedure and most of your findings should be the same.

Minor comments

- The authors introduce some abbreviations twice, i.e., AIMS and ACGME. You might don’t want to use abbreviations in the abstract.
- The conclusion is very short. You might want to address aspects you have not considered but are from interest.

We hope the authors regard our feedback as helpful for improving the paper. Furthermore, we suggest extending your team by a researcher more familiar with operations research to improve your scheduling part. We are pretty sure that some qualified persons exist at your university (maybe in industrial engineering or business).
