# Supplementary material for: Implementation of an automated scheduling tool improves schedule quality and resident satisfaction
Source: PLoS One. 2020 Aug 11;15(8):e0236952. doi: 10.1371/journal.pone.0236952 (PMC7418963; doi:10.1371/journal.pone.0236952)
Supplement: Attachment — Submitted filename: Response to reviewers 2.docx [file pone.0236952.s007.docx]

Response to reviewers:

Reviewer #2: We think the authors revised their paper very well. Our comments have all been taken into account. The revision of the literature has been very successful, and the paper can now generally be read more smoothly. So in our view, there is nothing to be said against accepting the paper for publication.

Minor comments
- p. 5, line 89: You should use “constraints” rather than “restraints”.
- p. 6, line 103: You might want to use “trainee” rather than “learner”.

**Response: Thank you for taking the time to review our paper and for your comments; we have made the recommended changes.**

Reviewer #3: The autors improved the manuscript. I like the revision, and have only a minor comment.

I would rather have the reference within the sentence it belongs to, e.g., "...pathway to independent practive [1]." instead of "...independent practive. [1]". However, please leave a space before the reference (unlike e.g., on page 9, references [20] and [9].

Thanks for the opportunity to review your paper!

**Response: Thank you for taking the time to review our paper and for your comments; we have made the recommended changes in citation formatting.**
